# Cation/Ca^2+^ Exchanger 1 (MdCCX1), a Plasma Membrane-Localized Na^+^ Transporter, Enhances Plant Salt Tolerance by Inhibiting Excessive Accumulation of Na^+^ and Reactive Oxygen Species

**DOI:** 10.3389/fpls.2021.746189

**Published:** 2021-10-13

**Authors:** Jie Yang, Weihan Li, Xin Guo, Peihong Chen, Yunpeng Cheng, Ke Mao, Fengwang Ma

**Affiliations:** State Key Laboratory of Crop Stress Biology for Arid Areas/Shaanxi Key Laboratory of Apple, College of Horticulture, Northwest A&F University, Yangling, China

**Keywords:** *Malus domestica*, salt tolerance, cation/Ca^2+^ exchanger 1, Na^+^ accumulation, ROS scavenging

## Abstract

High salinity causes severe damage to plant growth and significantly reduces crop yields. The CCX family proteins can facilitate the transport of multiple ions to prevent toxicity. CCX proteins play an important role in regulating plant salt tolerance, but no detailed studies on CCX proteins in apples have been reported. Here, the CCX family gene *MdCCX1* was cloned from apple (*Malus domestica*). It is constitutively expressed in various apple tissues and is significantly induced by salt stress. As a plasma membrane-localized protein, *MdCCX1*-overexpression could complement the Na^+^-sensitive phenotype of yeast mutants and reduce the Na^+^ content in yeast cells under NaCl treatment, suggesting that MdCCX1 could be a plasma membrane-localized Na^+^ transporter. To identify the function of *MdCCX1* in salt response, we transformed this gene into *Arabidopsis*, apple calli, and apple plants. Overexpression of *MdCCX1* significantly improved the salt tolerance of these transgenic materials. The significantly reduced Na^+^ content under NaCl treatment indicated that *MdCCX1* overexpression could enhance plant salt tolerance by inhibiting the excessive accumulation of Na^+^. Besides, *MdCCX1* overexpression could also enhance plant salt tolerance by promoting ROS scavenging. These findings provide new insight and rich resources for future studies of CCX proteins in plant species.

## Introduction

Land salinization is increasingly detrimental to plant reproduction and agricultural productivity, as most plant species are sensitive to high sodium concentrations. High salinity can lead to osmotic stress damaging multiple physiological and biochemical processes of plants. Plant salt tolerance is a complex trait that involves multiple physiological and biochemical mechanisms and numerous genes. Understanding the genetic and molecular mechanisms underlying salt tolerance is necessary for resolving such problems.

Plants have evolved a variety of mechanisms to deal with high salinity. Theoretically, three mechanisms can be used to prevent excess Na^+^ accumulation in plants (Keisham et al., 2018). First, Na^+^ entry into plant root cells may be restricted by ion channels such as non-selective cation channels (NSCCs) and transporters such as HAK5 and HKTs. Second, Na^+^ that enters the cells can be transported and stored in the vacuoles. Vacuolar compartmentation is an efficient strategy for plant cells to deal with salt stress, benefiting the osmotic adjustment. For example, halophytes naturally adapted to high salinity are known to accumulate large amounts of Na^+^ in the vacuole (Barros et al., 2021). Similar patterns for Na^+^ compartmentation by vacuolar Na^+^/H^+^ antiporters were found in *Arabidopsis* and tomato (Yokoi et al., 2002; Galvez et al., 2012). Third, cytosolic Na^+^ may be exported back to the growth medium or apoplastic spaces. Na^+^/H^+^ antiporters on the plasma membrane are expected to fulfill this function, such as SOS1 (Olias et al., 2009; Nie et al., 2015; Che et al., 2019).

Ion balance regulation, especially Na^+^, is related to various genes, including *SOS1*, *NHX1*, *HKT1*. Studies have shown that the cation/Ca^2+^ exchanger (CCX) family proteins are involved in Na^+^ regulation. The CCX proteins belong to the CaCA (Ca^2+^/cation antiporters) superfamily, which is conserved from bacteria to higher plants and animals (Pittman and Hirschi, 2016). Proteins in the CaCA superfamily usually facilitate the Ca^2+^ efflux against the concentration gradient across the membrane and promotes an influx of monovalent cations like H^+^, Na^+^, or K^+^ in exchange (Amagaya et al., 2019). Based on their function and evolutionary relationships, the CaCA proteins are divided into five different families: YRBG, Na^+^/Ca^2+^ exchanger (NCX), Na^+^/Ca^2+^, K^+^ exchanger (NCKX), cation/Ca^2+^ exchanger (CCX), and H^+^/cation exchanger (CAX) (Taneja et al., 2016; Amagaya et al., 2019; Mao et al., 2021). Although these proteins have a common conserved Na_Ca_ex structural domain (PF01699), diverse structural and functional characteristics differentiate them from one another. Only the CAX and CCX subfamilies exist in higher plants, with the two additional subgroups MHX (Mg^2+^/H^+^ exchanger) and NCL (NCX-like) (Cai and Lytton, 2004).

The CCX family proteins were identified in multiple eukaryotic organisms including protozoa, vertebrates, fungi, and plants (Cai and Clapham, 2012; Emery et al., 2012). Normally 3-6 CCX proteins are reported in land plants, including eight in *Glycine max* (Emery et al., 2012; Singh et al., 2014). These proteins contain two highly conserved α1 and α2-repeat regions. However, phylogenetic analysis classified CCX proteins into three subgroups, indicating a functional variation in CCX family proteins. In *Arabidopsis*, there are five CCX proteins (Pittman and Hirschi, 2016). AtCCX1 was proved to be an H^+^-dependent Na^+^/K^+^ exchanger that located on the vacuolar membrane of yeast. It increases Na^+^ accumulation and decreases K^+^ accumulation in the vacuole of *AtCCX1*-transformed *Pitch pastoris* GS115 (Chen et al., 2011). In *Arabidopsis*, *AtCCX1* overexpression accelerated natural and H_2_O_2_-induced senescence in leaves, while *ccx1ccx4* double mutant exhibited a stay-green phenotype by mediating Ca^2+^ signaling and reactive oxygen species (ROS) homeostasis (Li et al., 2016). AtCCX2 is an endoplasmic reticulum (ER) membrane-localized protein that regulates both cytosolic and ER [Ca^2+^], thus improves plant growth under salt stress (Corso et al., 2018). Its homologous gene *OsCCX2* in rice inhibits the sensitivity of the K667 yeast mutant to Ca^2+^. The *OsCCX2* transformed K667 yeast strain also showed enhanced tolerance toward excess Na^+^, Li^+^, Fe^2+^, Zn^2+^, and Co^2+^, suggesting its ability to transport both mono and divalent cations (Yadav et al., 2015). In another study, *OsCCX2* was found highly expressed in rice nodes. As a plasma membrane located transporter, OsCCX2 transports Cd^2+^ out of the cell and participates in root-to-shoot translocation of Cd^2+^ (Hao et al., 2018). AtCCX3 is an endomembrane-localized H^+^-dependent K^+^ transporter with apparent Na^+^ and Mn^2+^ transport activity (Morris et al., 2008). AtCCX4 could also suppress the defective phenotype of yeast mutants in Na^+^, K^+^, and Mn^2+^ transport (Morris et al., 2008). As to AtCCX5, ion transport characterization of this protein in yeast showed that it participates in high-affinity K^+^ uptake and Na^+^ transport (Zhang et al., 2011).

As an economically important fruit tree, the apple (*Malus* × *domestica* Borkh.) is one of the world’s most widely grown species. Its production is affected by various environmental stresses, including drought, cold, and salinity. Although CCX proteins play important roles in regulating plant resistance to salt stress, no detailed functional studies of CCX family proteins in apple have been reported untill now. To understand the *CCX* proteins’ function in regulating salt tolerance in apple, we cloned the CCX family gene *MdCCX1* from apple. Based on the *MdCCX1* functional characterization in transgenic yeast, *Arabidopsis*, apple calli, and apple plants, we proved that MdCCX1 could enhance plant salt tolerance by inhibiting excessive accumulation of Na^+^ and promoting the elimination of ROS. These results lay a foundation for further study of the mechanism of CCX proteins-mediated regulation of salt stress tolerance in apple plants.

## Materials and Methods

### Plant Materials, Growth Conditions, and Stress Treatments

The apple cultivar “Gala” (GL-3) was used to analyze *MdCCX1* expression under NaCl treatment. One-month-old plantlets with a consistent growth state were selected and treated with NaCl (200 mM) under hydroponic conditions (25°C, continuous white light) in Hoagland nutrient solution. Samples collected at specified time points were used to analyze the relative expression level of *MdCCX1*. RNA extraction and real-time quantitative RT-PCR analysis were performed as previously described (Mao et al., 2017). The malate dehydrogenase gene (*MdMDH*) was used as the endogenous control, and the relative expression level of *MdCCX1* was calculated with the 2^–△△CT^ method (Livak and Schmittgen, 2001). Three independent biological replications were performed for each experiment and four technical repetitions of each biological replicate were used.

*Arabidopsis thaliana* L. (Heyn), cv. Columbia (“Col”) and T_3_ generation transgenic seeds were used for NaCl treatment. Surface-sterilized seeds were sown on 1/2 MS agar medium containing 0, 100, 150, and 200 mM of NaCl and then placed at 4°C for vernalization. After two days of cultivation under light, seed germination rates were calculated and compared. Seedlings with fully emerged radicle tips (> 1 mm) were scored for seed germination rates. After cultured vertically on control medium (1/2 MS) for 5 d, all lines grow uniformly were plated on a 1/2 MS agar medium supplemented with 0, 50, 100, and 150 mM of NaCl for 6 days, and the primary root length and fresh weight of the different *Arabidopsis* seedlings lines were measured. For the phenotypic comparison of *Arabidopsis* plantlets under salt stress, four-week-old *Arabidopsis* with the same growing state were fully irrigated with 0 or 200 mM NaCl solution every 5 d for 15 d in a mixture of nutrient soil and perlite (1:1, v/v). Each experiment contains three independent replicates, each containing at least 24 plants.

For the salt treatment of calli, a 0.1 g portion of the wild-type (WT) and transgenic apple calli with similar growth states were cultured on a medium supplemented with 0, 100, 150, and 200 mM NaCl for 15 d. Each concentration treatment contained three plates as one replicate and 3 replicates in total.

For the salt treatment experiment on transgenic apple plants, the treatments were similar to those of *Arabidopsis*. Four-week-old apple seedlings were watered with 0 or 150 mM NaCl solution for 7 d.

### Bioinformatic Analysis of MdCCX1

Protein sequences of CaCA family members from *Arabidopsis* were downloaded from the TAIR database (The Arabidopsis Information Resource^1^). The phylogenetic tree of MdCCX1 and CaCA family proteins from *Arabidopsis* was constructed with MEGA 6.0 software (version 10.0.5; parameters setting: neighbor-joining method, bootstrap method, 1000 replicates, Poisson model, pairwise deletion). The prediction of conserved domains was performed using the full-length amino acid sequences of MdCCX1 and AtCCX1 using SMART^2^ and CD-Search^3^ softwares, respectively.

### Gene Cloning, Vector Construction, and Genetic Transformation of MdCCX1

Total RNAs were isolated from “Golden Delicious” apple leaves using Trizol reagent (Thermo-Fisher Scientific) with a CTAB-based method. cDNA synthesis was performed with a PrimeScript First-Strand cDNA Synthesis Kit (TaKaRa, Dalian, China). The sequence of *MD12G1011500* was downloaded from the GDR database (Genome Database for Rosaceae^4^), and gene-specific primers (*MdCCX1*-PMD-F and *MdCCX1*-PMD-R) were designed based on the mRNA sequence of *MD12G1011500*. Full-length coding sequence of *MdCCX1* was obtained through RT-PCR.

For *Arabidopsis* transformation, the resultant PCR product was inserted into the pBI121 vector under the control of the 35S promoter with *MdCCX1*-pBI121-F and *MdCCX1*-pBI121-R primers. Cloned vectors were genetically introduced into “Col” *Arabidopsis* by the *Agrobacterium tumefaciens* GV3101-mediated floral dip method (Clough and Bent, 1998). After kanamycin selection, PCR identification and qRT-PCR analysis, three homozygous transgenic lines with high *MdCCX1* expression were used in the follow-up experiments.

Apple calli (“Orin”) were transformed with the *MdCCX1*-pBI121 vector by the *Agrobacterium tumefaciens* EHA105-mediated method (Li et al., 2012). After kanamycin screening, calli cells differentiated from different positions (or from different dishes) in the culture medium were assigned to different lines and subcultured at 15 days intervals. After 5 serial subcultures, resistant calli showing stable growth were subjected to transgene PCR identification and qRT-PCR analysis for the *MdCCX1* expression level.

To obtain the composite plants with their roots genetically modified, the CDS of *MdCCX1* was cloned into pCambia2300-GFP vector with *MdCCX1*-2300GFP-F and -2300GFP-R primers, and a selected specific 300-bp fragment of *MdCCX1* was cloned into the RNAi vector pK7GWIWG2D, carrying a GFP tag for transformant selection. The resultant overexpression vector pCambia2300-*MdCCX1*-*GFP*, RNAi vector *MdCCX1*-RNAi-*GFP*, and two corresponding empty vectors were transferred into *Agrobacterium rhizogenes* K599 for genetic transformation of apple roots following the method previously reported (Xiang et al., 2005; Hernandez-Piedra et al., 2020). Briefly, one-month-old tissue cultured apple seedlings were cut off at the base of the stem with a surgical blade leaving about 1.5 cm long stem to create a diagonal incision. Stem and apex were immersed in the K599 suspension and vacuumed at 0.08 MPa for 15 min. After removing the excess bacterial fluid, the seedlings were transferred to the MS medium for further culture. When these seedlings grew new roots (about 1.5 months), we used a laser scanning confocal microscope (TCS SP8 SR) to identify roots with GFP fluorescence. qRT-PCR analysis identified the relative expression level of *MdCCX1* in the roots of these plants.

For transformation of yeast mutants, the CDS of *MdCCX1* was amplified with primers *MdCCX1*-pDR196-F/R and cloned into the pDR196 vector. The recombinant *MdCCX1*-pDR196 vector was transformed into following yeast strains using the LiAc/ss carrier DNA/PEG method (Gietz et al., 1995): Ca^2+^-sensitive yeast mutant K667 (Δ*cnb1*:LEU2 Δ*pmc1*:TRP1 Δ*vcx1*) (Cunningham and Fink, 1996) that was sensitive to high [Ca^2+^], the Na^+^-sensitive yeast mutants (Δ*ena1-4* and Δ*ena1-4*, Δ*nha1*) that were sensitive to high [Na^+^], R5421 (MATα *ura3-52 leu2 trk1*Δ *his3*Δ*200 his4-15 trk2*Δ*1:pCK64*) that was sensitive to low [K^+^], and wild type yeast strains W301-1B (MATα *leu2-3,112 trp1-1 can1-100 ura3-1 ade2-1 his3-11,15*) and BY4741 (MATa *his3*Δ*1 leu2*Δ*0 lys2*Δ*0 ura3*Δ*0*). After growth selection on selective medium (synthetic defined medium minus the appropriate amino acids) and transgene confirmation by PCR identification, two single colonies of each strain were selected for subsequent experiments.

Sequences of all primers used in this study are listed in Supplementary Table 1. Vectors were constructed using the Seamless Cloning/In-Fusion Cloning using One Step Cloning Kit (Vazyme Biotech Co., Ltd), Gateway BP Clonase II enzyme mix, and the Gateway LR Clonase Enzyme Mix (Invitrogen Gateway) following the manufacturers’ instructions.

### Identification of Ion Transport Function of MdCCX1 in Yeasts

Yeast transformants expressing *MdCCX1*-pDR196 or a control vector were selected and cultured in a yeast extract-peptone-dextrose (YPD) medium until OD_600_ = 0.6. For ion tolerance assays with different yeast strains, 1 ml of yeast culture were centrifuged and washed thrice with sterile water, 10 μl aliquots of each 10-fold serial dilution (10^0^, 10^1^, 10^2^, 10^3^, and 10^4^) were spotted onto YPD plates supplemented with different concentrations of CaCl_2_, LiCl, MnCl_2_, CdCl_2_, BaCl_2_, CuCl_2_, or MgCl_2_, and then incubated at 28°C for 3 d. K667 and K667 transformants expressing the empty vector pDR196 were used as negative controls, and W303-1B was used as a positive control. For Na^+^ treatments, the two Na^+^-sensitive mutants served as negative controls. For K^+^ treatment, R5421 positive transformants were spotted onto AP plates (no K^+^) supplemented with 0, 0.2, 0.5, 5, and 25 mM KCl. R5421 and R5421 transformants expressing the empty vector pDR196 were used as negative controls, and the BY4741 strain was used as a positive control.

To measure cell growth rate, yeast cells were diluted to 0.1 at OD_600_ and then cultured in the YPD medium supplemented with 200 mM NaCl. The concentration was measured within 8 h at 1-h intervals.

To determine Na^+^ content in yeast, the initial concentration of positive transformants of BY4741 and control strains was adjusted to 0.1. Yeast cells of an equal volume were collected and cultured in YPD liquid medium supplemented with 0, 100, 200, and 300 mM NaCl until the logarithmic phase. Yeast cells were then collected for determination of sodium content.

### Subcellular Localization Analysis of MdCCX1

The pCambia2300-*MdCCX1*-*GFP* and pCambia2300-*AtCBL1*-*mCherry* were introduced into the *Agrobacterium tumefaciens* strain GV3101, respectively. After PCR identification, the two transgenic *Agrobacterium* strains and p19 strain (a helper strain) were cultured overnight until OD_600_ = 0.6, collected and adjusted OD_600_ = 1.5 with a resuspension solution (10 mM MES, 10 mM MgCl_2_, 150 μM acetosyringone, pH5.8). The three bacterial solutions were mixed at a volume ratio of 1:1:1 so that the final concentration of each strain was OD_600_ = 0.5. After incubation at room temperature for 2–4 h, the mixture was used to infiltrate the leaves of 4–6 weeks old tobacco plants (*Nicotiana benthamiana*) through a 1 ml syringe. These plants were then cultured for 3 d under a 12 h light/12 h dark photoperiod at 25°C for incubation. The AtCBL1 (AT4G17615) served as a plasma membrane-localized marker.

*Arabidopsis* protoplasts isolation and transformation were performed as reported previously (Damm et al., 1989; Karesch et al., 1991). Briefly, 4-week-old *Arabidopsis* 4-7th rosettes leaves were digested overnight with a mixture solution of macerozyme R-10 (Yakult Pharmaceutical Industry Co., Ltd.) and cellulose R-10 (Yakult Pharmaceutical Industry Co., Ltd.). The pCambia2300-*MdCCX1*-*GFP* and pCambia2300-*GFP* vectors were concentrated by ethanol, and transformed into protoplasts by the PEG-CaCl_2_-mediated method and incubated for 18 h under low light at 23°C.

All fluorescence images were obtained using a confocal laser scanning microscope (Leica TCS-SP8 SR). GFP fluorescence signals were detected at 500 to 535 nm after excitation at 488 nm, while mCherry was excited at 543 nm and scanned at 600–630 nm.

### Measurement of Physiological Parameters

Total chlorophyll content and relative electrolytic leakage (REL) were examined as described previously, respectively (Liang et al., 2017; Hang et al., 2020). Malondialdehyde (MDA) content and activities of CAT, POD, and SOD were measured according to the manufacturer’s instructions using detection kits (Suzhou Comin Biotechnology Co., Ltd., Suzhou, China). For ROS accumulation in the plant, leaves were covered with 0.1% (w/v) DAB for 12 h and 10 mM phosphate buffer (pH 7.8) containing 1 g/L NBT for 4 h at 28°C in the dark for detecting the production of H_2_O_2_ and O_2_^–^ by histochemical staining, respectively. The chlorophyll was removed from the soaked leaves by 96% (v/v) ethanol (Zhou et al., 2019). H_2_O_2_ and O_2_^–^ contents were determined according to the instructions of the kits (Suzhou Comin Biotechnology Co., Ltd., Suzhou, China). To demonstrate ROS accumulation level in *Arabidopsis* root tips, the roots of seedlings were incubated in 20 μM C-H2DCFDA solution for 30 min at room temperature in the dark. After washed off the excess reagents with 0.9% NaCl solution, green fluorescence images were obtained by confocal microscopy (Neverisky and Abbott, 2015).

Chlorophyll fluorescence after 30 min of dark adaptation was measured using Open FluorCam FC 800-O and analyzed with Fluorcam7 software (PSI, Brno, Czech Republic). The net photosynthetic rates were recorded by a portable photosynthesis system (Li-6400; LICOR, Huntington Beach, CA, United States) between 8:00 and 10:00 am with 1000 μmol photons⋅m^–2^⋅s^–1^ and a constant airflow rate of 500 μmol⋅s^–1^. The cuvette CO_2_ concentration was set to 400 μmol CO_2_ mol^–1^ air, with a vapor pressure deficit of 2.0–3.4 kPa.

### Determination of Na^+^ Concent

Plant materials or yeast cells were dried at 105°C for 0.5 h and then at 65°C for 3 d. After grinding into powder, 0.1 g of each sample was digested with concentrated HNO_3_ and H_2_O_2_ solutions for 60 min at 220°C using a muffle furnace. According to the previous method, Na^+^ concent was measured using a flame photometer (M410; Sherwood Scientific Ltd., Cambridge, United Kingdom).

### Statistical Analysis

IBM SPSS software (version 26; IBM, Chicago, Illinois, United States) was used to compare the differences via One-way ANOVA, Duncan’s test or Student’s *t-*test. Differences between means were deemed to be significant when the *P*-*value* of the test was less than 0.05.

## Results

### Gene Cloning, Sequence Analysis and Expression Patterns of *MdCCX1*

**FIGURE 1:**
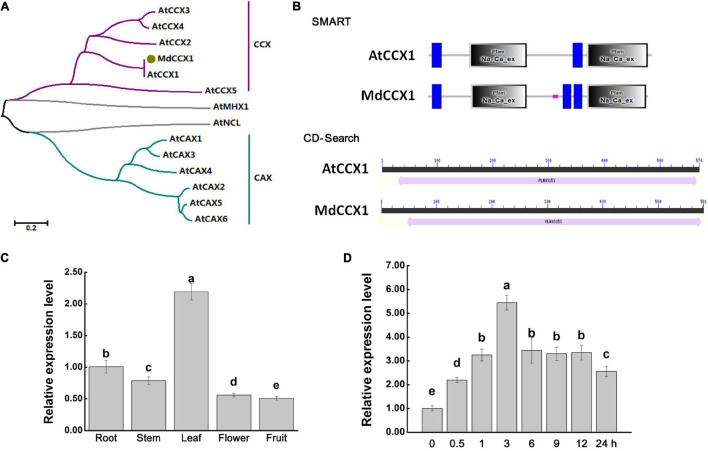
Protein sequence analysis and gene expression identification of *MdCCX1*. **(A)** Phylogenetic analysis of MdCCX1 and CaCA superfamily members in *Arabidopsis*. The phylogenetic was constructed with MEGA-X software. The scale bar represents 0.2 substitutions per site. **(B)** Conserved domains identified in AtCCX1 and MdCCX1 proteins using the online SMART database (http://smart.embl-heidelberg.de) and CD-Search tool (https://www.ncbi.nlm.nih.gov/Structure/cdd/wrpsb.cgi). **(C)** Relative expression levels of *MdCCX1* in different apple tissues. **(D)** Relative expression levels of *MdCCX1* in roots in response to 200 mM NaCl treatment. The relative expression levels of *MdCCX1* were calculated with respect to the control samples (root in C and 0 h in D) by the 2 ^–ΔΔCT^ method. *MdMDH* served as the reference gene. Different letters represent significant differences (One-way ANOVA analysis of variance and Duncan’s test: *p* < 0.05). Accession number: AtCCX1 (AT5G17860.1), AtCCX2 (AT5G17850.1), AtCCX3 (AT3G14070.1), AtCCX4 (AT1G54115.1), AtCCX5 (AT1G08960.1), AtCAX1 (AT2G38170.3), AtCAX2 (AT3G13320.1), AtCAX3 (AT3G51860.1), AtCAX4 (AT5G01490.1), AtCAX5 (AT1G55730.1), AtCAX6 (AT1G55720.1), AtMHX1 (AT2G47600.1), AtNCL (AT1G53210.1), MdCCX1 (MD12G1011500).

In our recent study, the gene *MD12G1011500* was identified as the *AtCCX1* ortholog in apple (Mao et al., 2021). Gene specific primers were designed based on *MD12G1011500* and used to amplify the full-length coding sequence (CDS) of *MdCCX1*. Using the total RNA extracted from leaves of “Golden Delicious” apple plants as a template, we obtained the CDS of *MdCCX1* by RT-PCR. The sequencing results showed that the CDS of *MdCCX1* was 1746 bp in length, encodes a protein of 581 amino acids, with a predicted mass weight of 63.27 kDa and a pI of 7.73. To investigate the phylogenetic relationship between MdCCX1 and the CaCA family members in *Arabidopsis*, a phylogenetic tree was constructed using their protein sequences (Figure 1A). MdCCX1 was clustered in the same branch with AtCCX1, indicating that MdCCX1 should be the ortholog of *AtCCX1* in apple. We also identified the conserved domains contained in AtCCX1 and MdCCX1, using the online CD-Search and SMART softwares. Results showed that MdCCX1 belonged to the cation/calcium exchanger superfamily (PLN03151 superfamily) and contained two conserved Na_Ca_ex domains, just like AtCCX1 (Figure 1B). These results further confirm that the cloned *MdCCX1* should be the ortholog of *AtCCX1* in apple.

Total RNA of roots, stems, leaves, flowers and fruits of “GL-3” apple plants were extracted and used for qRT-PCR analysis to investigate the expression pattern of *MdCCX1* in different apple tissues. The expression level of *MdCCX1* was highest in leaf, followed by root and stem, with the lowest levels in flower and fruit (Figure 1C). Previous studies had shown that CCX family genes were involved in salt tolerance regulation. To explore the response of *MdCCX1* to salt stress, “GL-3” seedlings were treated with 200 mM NaCl using a hydroponic system. Seedlings roots were collected after different time points of salt treatment and used for RNA extraction and qRT-PCR expression analysis. Results showed that the *MdCCX1* expression increased continuously and peaked at 3 h of salt treatment. Subsequently, although the expression level of *MdCCX1* continued to decrease, its expression level at the end of treatment was still significantly higher (2.5x) than that at 0 h (Figure 1D). This result proved that *MdCCX1* responded to salt stress significantly, especially in the early stage of the stress response, suggesting a role in regulating the salt stress response.

### Subcellular Localization of MdCCX1 Protein

**FIGURE 2:**
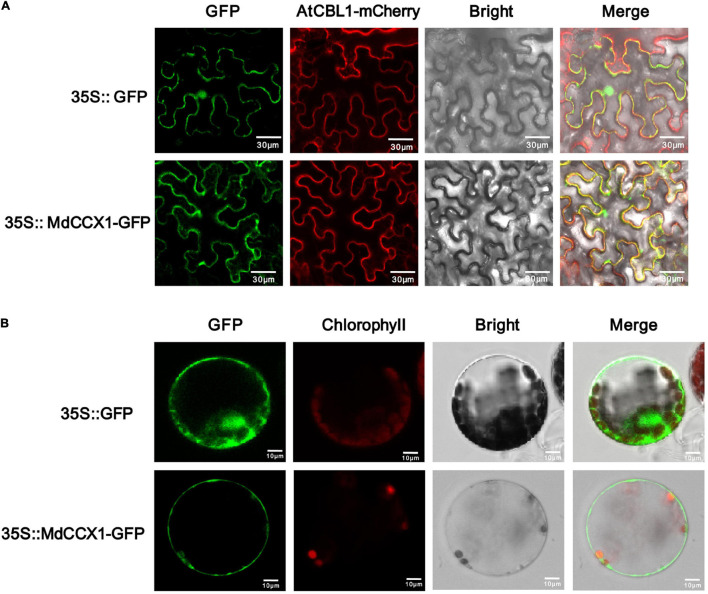
Subcellular localization of the MdCCX1 protein. **(A)** Fluorescence of the MdCCX1-GFP fusion protein in tobacco epidermal cells. The AtCBL1-mCherry fusion protein served as a plasma membrane-localized marker. **(B)** Fluorescence of the MdCCX1-GFP fusion protein in *Arabidopsis* protoplasts. The empty pCambia2300-GFP vector was used to express the GFP protein as a control. Fluorescence, bright field, and merged images of representative cells are shown. Scale bars, 30 μm in tobacco epidermal cells; 10 μm in *Arabidopsis* protoplasts.

The subcellular localization of ion transporters, such as CCX proteins, plays a crucial role in ion transport and regulation of stress response. The CDS of *MdCCX1* was cloned into the pCambina2300-GFP vector to determine the subcellular localization of MdCCX1. AtCBL1 fused with a mCherry tag was used as a cell membrane marker. Then, the 35S:*MdCCX1-GFP* and 35S:*AtCBL1-mCherry* constructs were transformed into the epidermal cells of tobacco (*Nicotiana benthamiana*) leaves by the *Agrobacterium*-mediated instantaneous expression method. After 3 days of culture, the GFP fluorescence in cells of tobacco leaves was observed under a fluorescence microscope. When the GFP control vector was expressed, the GFP fluorescence could be observed ubiquitously throughout the cell. However, in MdCCX1-GFP expressing cells, the green fluorescence signal was localized on the cell membrane and colocalized with the red fluorescence signal of AtCBL1-mCherry (Figure 2A). To further confirm the subcellular localization of MdCCX1 in plants, we also expressed the GFP control vector and 35S:*MdCCX1-GFP* construct in *Arabidopsis* protoplasts. The green fluorescence signal of the GFP control was distributed ubiquitously throughout the protoplast, whereas the MdCCX1-GFP fusion protein could be observed only on the plasma membrane of the protoplast cell (Figure 2B). These results suggested that MdCCX1 localized on the plasma membrane of plant cells.

### Analysis of Ion Transport Characteristics of MdCCX1 in Calcium and Sodium-Sensitive Yeast Mutants

**FIGURE 3:**
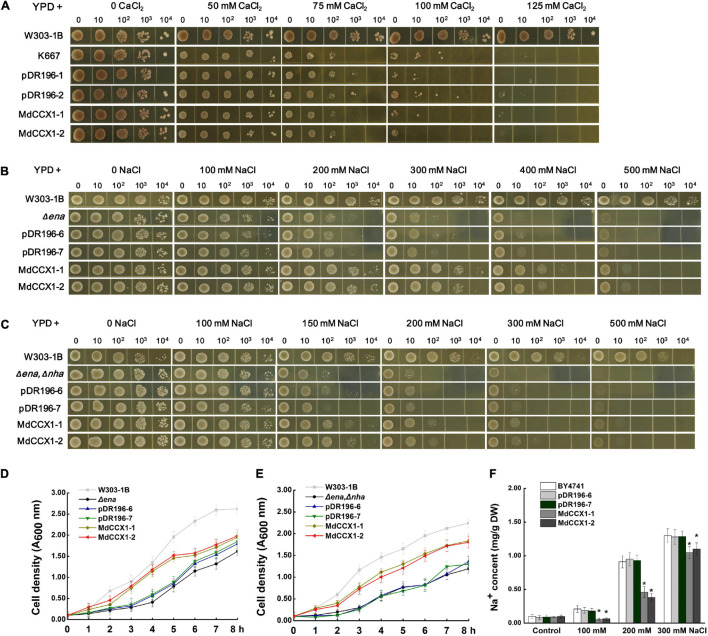
Functional complementarity experiments of *MdCCX1* in yeast mutants. **(A)** Identification of Ca^2+^ transport characteristics of MdCCX1 in K667. The K667 yeast triple mutant (Δpmc1Δvcx1Δcnb1) was transformed with the pDR196 empty vector or pDR196-*MdCCX1*. 10-μl aliquots of serial dilutions (10, 10^2^, 10^3^, and 10^4^) were dotted onto YPD medium in the presence of 0, 50, 75, 100, 125 mM CaCl_2_ and grown for 3 d. W303-1B was the positive control. **(B)** and **(C)** Na^+^ tolerance of yeast cells expressing pDR196-*MdCCX1* or pDR196 in Δ*ena1-4* **(B)** or Δ*ena1-4*, Δ*nha1* **(C)** yeast mutants. After gradient dilution of the positive transformants and the control yeast lines, 10-μl aliquots of serial dilutions (10, 10^2^, 10^3^, and 10^4^) were dotted onto YPD medium supplemented with different concentrations of NaCl and grown for 3 d. **(D)** and **(E)** Growth curves of yeast strains in **(C)** and **(D)** under 200 mM NaCl treatment with an initial concentration of OD 600 = 0.1. **(F)** Sodium content in yeast cells. The wild-type yeast BY4741 was transformed with pDR196-*MdCCX1* or pDR196. All yeast strains grown in YPD medium supplemented with 0, 100, 200, or 300 mM NaCl to the logarithmic growth phase were collected for Na^+^ determination. Data are average values of three independent experiments and are presented as mean ± SD. Significant differences (*) relative to the empty vector and BY4741 were determined using Student’s *t-test*: *p* < 0.05.

Yeast mutants sensitive to specific ions provide convenience to study the ion transport characteristic of various transporters. K667 yeast strain, which is sensitive to high [Ca^2+^], is often used to identify the Ca^2+^ transport characteristic of CaCA superfamily proteins. To test whether the MdCCX1 protein could transport Ca^2+^, the full-length CDS of *MdCCX1* was cloned into the yeast expression vector pDR196 for the genetic transformation of K667. K667 yeast mutant and positive transformants of empty vector pDR196 were used as negative controls. These yeast strains were diluted in series and cultured in a YPD medium supplemented with different CaCl_2_ concentrations for growth comparison. To our surprise, the growth of the *MdCCX1*-transformed yeast strains showed no noticeable difference compared with the negative controls (Figure 3A). This result suggested that the MdCCX1 does not have calcium ion transport capacity, which differs from CCX proteins in *Arabidopsis* and rice. In addition to Ca^2+^, studies in rice have shown that OsCCX2 could also transport various monovalent and divalent ions. Thus, we further tested whether MdCCX1 could transport other ions containing Li^+^, Mn^2+^, Cd^2+^, Ba^2+^, Cu^2+^, and Mg^2+^ using the K667 strain. Unfortunately, *MdCCX1*-overexpression did not enhance the tolerance of transgenic yeast strains to these ions, suggesting that MdCCX1 should not be able to transport these ions either (Supplementary Figure 1A).

To investigate whether *MdCCX1* has Na^+^ transport capacity, two sodium-sensitive yeast mutant strains, the Δ*ena1-4* mutant that lost the Na^+^-ATPase on the cell membrane and the Δ*ena1-4*,Δ*nha1* double mutant that lost the Na^+^-ATPase and Na^+^/H^+^ exchanger on the cell membrane, were used. We firstly overexpressed the *MdCCX1* in the Δ*ena1-4* mutant yeast strain, with the pDR196 used as a negative control. Under the YPD medium condition (without NaCl), there was no growth difference between *MdCCX1*-transformed and pDR196 control strains. However, at high NaCl concentration, the growth of all strains was significantly inhibited, and the inhibition effect of the control strains was more significant, especially at 200-, 300-, and 400 mM NaCl (Figure 3B). Similar results were found in the Δ*ena1-4*,Δ*nha1* double mutant yeast strain (Figure 3C). To further confirm these results, we measured the growth rates of these strains cultured in YPD liquid medium supplemented with 200 mM NaCl. The results showed that the growth rates of the *MdCCX1*-overexpressing transformants were significantly faster than that of the pDR196 control strains (Figures 3D,E), suggesting that *MdCCX1-*overexpression enhanced the salt tolerance of transgenic yeast strains. This could be due to restoring the loss of sodium efflux function in the two yeast mutants.

The missing *ENA* gene in these sodium-sensitive yeast mutants encods a P-type ATPase responsible for Na^+^ excretion and K^+^ selective absorption. To rule out the effect of K^+^, the R5421 yeast mutant that sensitive to low [K^+^] (deficient in K^+^ absorption) was used to identify whether the MdCCX1 protein could promote K^+^ absorption. We transformed the *MdCCX1*-pDR196 and pDR196 emptor vector into the R5421 strain, respectively. Then the transgenic strains were cultured on the AP medium (potassium free) supplemented with different KCl concentrations. Results showed that overexpression of *MdCCX1* could not suppress the sensitivity to low K^+^ concentration of R5421, suggesting that MdCCX1 did not have the K^+^ absorption capacity (Supplementary Figure 1B).

To identify whether MdCCX1 could promote the efflux of Na^+^ in yeast cells, *MdCCX1* was transformed into the yeast strain BY4741. The transformants were incubated in YPD liquid medium containing 0, 100, 200, and 300 mM NaCl until the OD_600_ value reached 0.6. The yeast cells were then collected to determine the Na^+^ content. As shown in Figure 3F, the Na^+^ content of *MdCCX1*-overexpressing yeast cells was significantly lower than that of control strains, suggesting that MdCCX1 should have the Na^+^ efflux transport activity.

### Ectopic Expression of *MdCCX1* Promoted Seed Germination and Enhanced Salt Tolerance in Transgenic *Arabidopsis*

**FIGURE 4:**
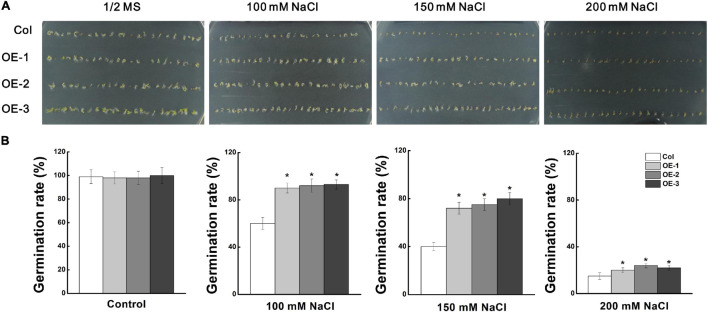
Analysis of seed germination rate of “Col” and *MdCCX1*-overexpressing transgenic lines under NaCl stress. **(A)** Typical picture of germination rate. T_3_ seeds from *MdCCX1*-overexpressing lines were dispersed on 1/2 MS medium containing different concentrations of NaCl (0, 100, 150, and 200 mM) or not for 2 d. **(B)** Statistics of germinated seeds in **(A)**. Data are average values of three independent experiments and are presented as mean ± SD. Significant differences (*) relative to the “Col” were determined using Student’s *t-test*: *p* < 0.05.

To investigate the role of *MdCCX1* in regulating plant salt tolerance, it was ectopically expressed in *Arabidopsis*. Through kanamycin resistance selection, PCR identification and *MdCCX1* expression analysis (Supplementary Figures 2A,B), the T_3_ generation seeds of three representative lines with high expression levels of *MdCCX1* (OE-1, OE-2, OE-3) were selected for subsequent salt treatment and phenotypic analysis. Firstly, we measured the seed germination rates of these transgenic lines and Col. The seeds were vernalized for 3 days at 4°C and then sown in the 1/2 MS medium containing 0, 50, 100, and 150mM NaCl. After 2 days of culture, we counted the germination rate of each line. Col and *MdCCX1-*overexpressing lines on 1/2 MS medium showed no significant difference in germination, with the germination rate of all lines closed to 100% (Figure 4). The addition of NaCl decreased the seed germination rates of all lines, but that of the *MdCCX1-*overexpressing lines were significantly higher than Col (Figure 4), suggesting that overexpression of *MdCCX1* alleviated the inhibition effect of salt stress on seed germination.

**FIGURE 5:**
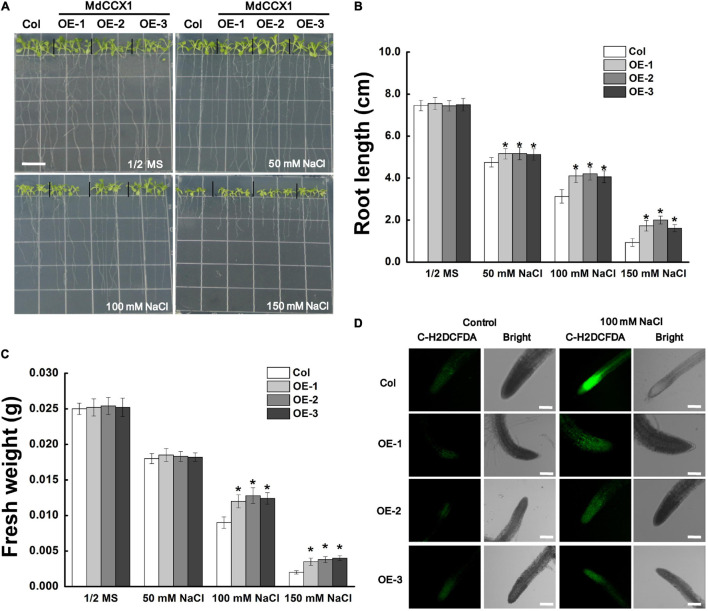
Phenotypic comparison of “Col” and *MdCCX1*-overexpressing transgenic *Arabidopsis* seedlings under NaCl stress. **(A)** Representative images of five-day-old “Col” and transgenic seedlings had been cultivated for 6 d on 1/2 MS medium supplemented with 0, 50, 100, and 150 mM NaCl. Bars = 1 cm. Root length **(B)** and fresh weight **(C)** of whole seedling in **(A)**. Error bars represent SD based on 3 independent replicates. **(D)** Accumulation of ROS in root tips treated with 100 mM NaCl in **(A)**. Roots were labeled with the ROS-sensitive fluorescent probe C-H2DCFDH. The bright and fluorescence fields are represented. Scale bars, 100 μm. For **(B,C)**, bars labeled with * in each panel indicates values that are significantly different relative to “Col” at *p* < 0.05, using Student’s *t-test*.

Next, we studied the effect of salt stress on the growth of *Arabidopsis* seedlings. 5-day-old *Arabidopsis* seedlings were placed vertically on 1/2 MS medium supplemented with 0, 50, 100, and 150 mM NaCl and cultured under long-day conditions for 6 days. In normal 1/2 MS medium, there was no noticeable difference on root length and fresh weight between *MdCCX1* transgenic lines and Col seedlings. However, under NaCl treatments, the root length of these *MdCCX1* transgenic lines was significantly longer, and the fresh weight was significantly higher than that of the Col seedlings (Figures 5A–C). Salt stress could trigger the excessive accumulation of ROS, resulting in oxidative damage to plant tissues. Here, C-H2DCFDA fluorescence dye was used to visualize the ROS accumulation in the seedlings’ roots. Under normal conditions, OE lines and the Col showed weak fluorescence signals in root tips, indicating a low level of ROS accumulation. Under 100 mM NaCl, the fluorescence in root tips of all lines was stronger than that of the control group, while the fluorescence of *MdCCX1* transgenic lines was significantly weaker than the Col seedlings, indicating that Col seedlings accumulated more ROS in roots under salt stress (Figure 5D). These results suggested that overexpression of *MdCCX1* enhanced the salt tolerance of transgenic *Arabidopsis* seedlings.

**FIGURE 6:**
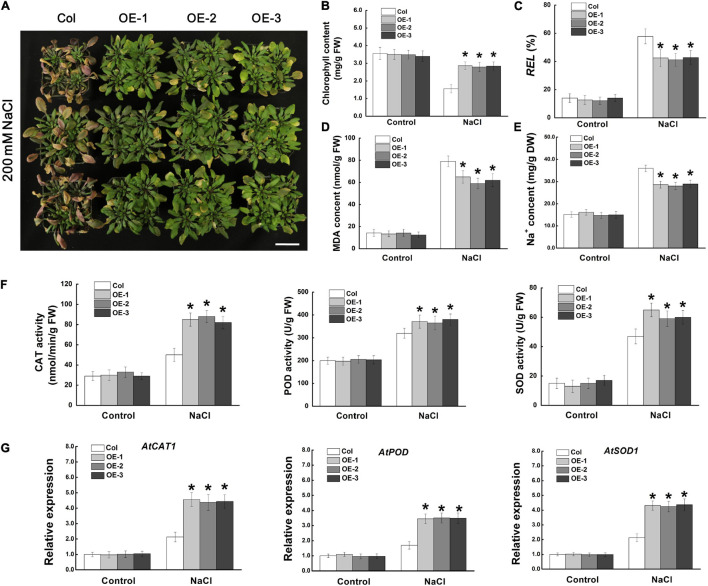
Assessment of salt tolerance in “Col” and *MdCCX1*-overexpressing transgenic *Arabidopsis* plants. **(A)** Representative images of “Col” and transgenic plants exposed to 200 mM NaCl for 15 d. Scale bars, 4 cm. Total chlorophyll content **(B)**, relative electrolyte leakage **(C)**, MDA content **(D)**, and Na^+^ content **(E)** of all seedlings lines measured after NaCl treatment. **(F)** Enzymatic activity of antioxidant enzymes CAT, POD, and SOD measured after NaCl treatment. **(G)** The relative expression levels of *AtCAT1*, *AtPOD*, and *AtSOD1*. *AtActin* served as a reference gene. Error bars represent SD based on 3 independent replicates. For **(B–G)**, bars labeled with * in each panel indicates values that are significantly different from “Col” at *p* < 0.05, using Student’s *t-test*.

We further investigated the regulatory function of *MdCCX1* on salt tolerance using one- mouth-old adult *Arabidopsis* plants grown in soil. Under normal conditions, there was no noticeable morphological difference between the transgenic lines and Col plants. However, after 14 days of NaCl treatment, most of the leaves of the Col plants turned yellow, wilted, or even died, indicating that these plants had suffered severe stress damage. However, most of the leaves of the *MdCCX1* transgenic plants remained green and vigorous, and only the edge of some leaves showed a yellowing phenotype (Figure 6A). Measurements of stress-related physiological indices, such as chlorophyll content, relative electrolyte leakage (REL), and MDA levels, also support the phenotypic differences. Under normal conditions, there was no significant difference in physiological indices between *MdCCX1* transgenic lines and control plants. However, under salt treatment, the chlorophyll content was significantly higher, and the REL and MDA content was significantly lower in *MdCCX1* transgenic lines than in the control plants (Figures 6B–D). These results indicated that overexpression of *MdCCX1* enhanced the salt tolerance of transgenic *Arabidopsis* plants.

Ion transporters could enhance plant salt tolerance by expelling extra Na^+^ from the cell or by compartmentalizing them into vacuoles to prevent Na^+^ toxicity. Based on the function of *MdCCX1* in promoting the efflux of Na^+^ in yeast cells (Figure 3), we measured the Na^+^ content in these NaCl-treated *Arabidopsis* plants. Under normal conditions, all plants kept at basal Na^+^ content (∼15.1 mg/g DW), with no difference between different lines. However, after NaCl treatment, the Na^+^ content in the three transgenic lines was significantly lower than that of the control plants (Figure 6E). This result indicated that *MdCCX1*-overexpression enhanced transgenic plants’ salt tolerance by inhibiting the excessive accumulation of Na^+^ under salt stress.

Stress-induced excessive accumulation of ROS is harmful to plants. The antioxidant enzymes such as catalase (CAT), peroxidase (POD), and superoxide dismutase (SOD) are key enzymes for ROS scavenging. Thus, the activities of these three antioxidant enzymes were examined. Under control conditions, the CAT, POD and SOD enzymatic activities were similar in all plants. However, the transgenic seedlings showed stronger antioxidant enzyme activities than Col plants under salt stress (Figure 6F), suggesting that *MdCCX1*-overexpression increased the antioxidant enzymes’ activity in response to salt treatment. To further study how *MdCCX1*-overexpression increases the activity of these antioxidant enzymes, the expression levels of genes encoding these enzymes were examined. Consistent with the enzyme activity, the expression levels of *AtCAT1*, *AtPOD*, and *AtSOD1* were also significantly higher in *MdCCX1* transgenic plants under salt stress (Figure 6G). These results indicated that in addition to inhibiting Na^+^ accumulation, MdCCX1 could also enhance plant salt tolerance by promoting the activity of antioxidant enzymes.

### Overexpression of *MdCCX1* Improved Salt Tolerance of Transgenic Apple Calli

**FIGURE 7:**
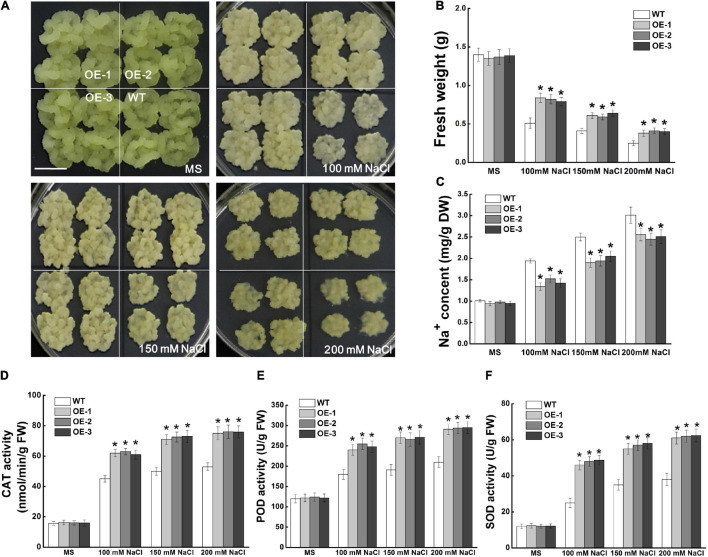
Effects of NaCl treatment on the growth of wild type and *MdCCX1*-overexpressing transgenic apple calli. Representative images **(A)**, fresh weight **(B)**, Na^+^ content **(C)**, and activity of antioxidant enzymes **(D–F)** in WT and transgenic apple calli. The apple calli was cultured on MS medium supplemented with 0, 100, 150, and 200 mM NaCl for 15 d. Scale bars, 1 cm. For **(B–F)**, bars labeled with * in each panel indicate values that are significantly different form the WT at *p* < 0.05, using Student’s *t-test*.

*MdCCX1*-pBI121-transformed apple calli was used to identify the function of *MdCCX1* in regulating salt tolerance in apple. Through PCR identification and expression analysis, three independent transgenic lines with high *MdCCX1* expression levels were selected (Supplementary Figures 2C,D). *MdCCX1* transgenic and wild-type apple calli (WT) were grown on MS medium supplemented with different concentrations of NaCl (0, 100, 150, and 200 mM). After 15 days of culture on a normal MS medium, there was no significant growth difference between *MdCCX1*-overexpression transgenic lines and WT (Figure 7A). NaCl inhibited the WT and transgenic lines growth, but the growth rate of transgenic lines was significantly faster than that of WT (Figure 7A). Consistent with the phenotypic phenomenon, the fresh weight of the transgenic lines was also significantly higher than that of the WT (Figure 7B). These results suggested that overexpression of *MdCCX1* decreased the inhibition effect of salt stress on apple calli growth.

We measured the Na^+^ content of apple calli cultured under normal and salt-treated conditions. With the increase of NaCl concentration, the Na^+^ content of all lines of apple calli increased, but the Na^+^ contents of *MdCCX1*-overexpression transgenic calli were significantly lower than that of WT (Figure 7C). This suggested that *MdCCX1* could enhance the salt tolerance of apple calli by inhibiting Na^+^ accumulation. Because *MdCCX1*-overexpression could improve the activity of antioxidant enzymes in transgenic *Arabidopsis* under salt stress (Figure 6F), we further investigated the effect of MdCCX1 on the antioxidant enzyme activity in apple calli. As we expected, under salt stress, the antioxidant enzyme activity in MdCCX1 transgenic calli was significantly higher than that of the wild type (Figures 7D–F), suggesting that MdCCX1 could improve salt tolerance of apple calli by promoting antioxidant enzyme activity.

### *Agrobacterium rhizogenes*-Mediated Transformation of *MdCCX1* in Apple Roots Affected Salt Tolerance of Apple Plants

**FIGURE 8:**
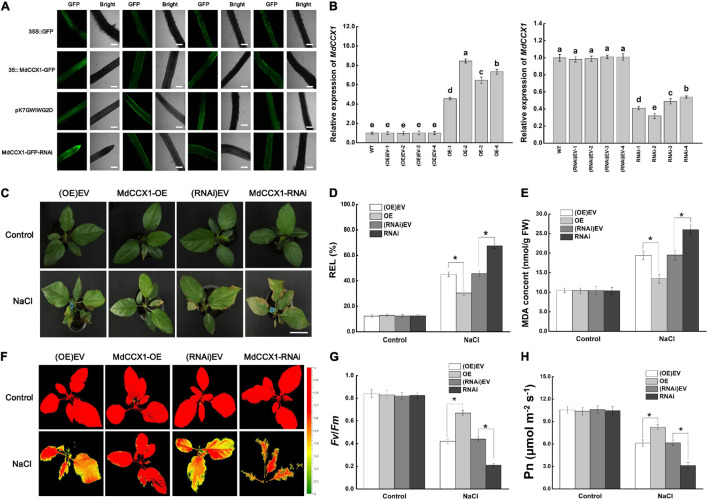
Phenotypic analysis of transgenic apple plants under 150 mM NaCl treatment. **(A)** Representative images of GFP fluorescence of transgenic apple roots. OE-EV and RNAi-EV represent roots transformed with the empty vector pCambia2300 or pK7GWIWG2D. Scale bars, 300 μm. **(B)** Identification of *MdCCX1* expression in transgenic apple roots. **(C)** Typical images of phenotypes treated with 0 or 150 mM NaCl for 7 d. Scale bars, 4 cm. **(D)** Relative electrolyte leakage. **(E)** MDA content. Representative chlorophyll fluorescence images **(F)** and Fv/Fm ratios **(G)** of different transgenic lines under normal and NaCl stress conditions. The false colors in the images indicate the Fv/Fm ratios ranging from 0 (black) to 1.0 (red). **(H)** Net photosynthetic rate (Pn). The values of each index are the average values from all lines in transgenic plants of the same type for **(D)**, **(E)**, **(G)**, and **(H)**. Values are means of 20 replicates ± SD. * in each panel indicates values that are significantly different from the corresponding control lines at *p* < 0.05, using Student’s *t-test*.

*A. rhizogenes*, a soil pathogen that elicits adventitious, genetically transformed roots is used for obtaining transgenic roots. *A. rhizogenes* leads to the production of so-called “composite plants” comprising a transgenic hairy root system attached to non-transformed shoots. Since we have not yet obtained the stable transgenic apple plants in which *MdCCX1* is overexpressed or disturbed, this approach facilitates the rapid study of its function in regulating salt tolerance in apples. First, the coding region and a specific inhibitory fragment of *MdCCX1* were cloned into the vectors pCambia2300-GFP and pK7GWIWG2D-GFP, respectively. Then apple plants with transgenic hairy roots were generated from “GL-3” by *A. rhizogenes* K599-mediated transformation method. GFP fluorescence showed that the vectors were successfully expressed in roots (Figure 8A). Expression analysis showed that *MdCCX1* was upregulated in roots of *MdCCX1*-overexpressing (OE) lines but noticeably downregulated in RNAi lines compared to empty vector controls (Figure 8B). No significant differences were observed in the expression of *MdCCX1* between the empty vector control lines (OE-EV and RNAi-EV) and WT (Figure 8B).

Apple plants were subsequently fully irrigated with 0 (control group) or 150 mM NaCl solution for salt treatment. Under normal conditions, there were no significant differences between different transgenic lines. However, after 7 days of salt treatment, the MdCCX1-OE plants exhibited a better growth state than the (OE)-EV lines, with their leaves still bright green and vigorous. By contrast, down-regulation of *MdCCX1* expression made the apple plants grow worse than the (RNAi)-EV lines, with more brown spots and areas appears in their leaves (Figure 8C). DAB and NBT histological staining results of leaves also indicated a high accumulation level of H_2_O_2_ and O_2_^–^ (Supplementary Figure 3). We also measured the stress-related physiological indices of REL and MDA content in leaves of these plants to evaluate the damage caused by salt treatment. Under salt stress, the REL and MDA content were significantly lower in the leaves of MdCCX1-OE plants but significantly higher in MdCCX1-RNAi plants (Figures 8D,E). These results suggested that overexpression of *MdCCX1* in roots significantly enhanced the salt tolerance of apple plants.

Chlorophyll fluorescence is an appropriate tool for early identification of the degree of damage in plants. Maximal photochemical efficiency of PS II in the dark (Fv/Fm) was an important indicator of chlorophyll fluorescence parameters, so we measured the fluorescence of each line in both control and NaCl treatment groups. The Fv/Fm ratios of all lines in the control group were similar and maintained at a high level (Figures 8F,G). However, under salt treatment, the Fv/Fm ratios of the MdCCX1-OE lines were significantly higher than the two control lines, whereas that of the MdCCX1-RNAi lines were significantly lower than the control lines (Figures 8F,G). Damage to photosynthetic units directly affected photosynthesis. Thus, we further measured the net photosynthetic rate (Pn) of these plants. Results showed that the performance of Pn in these plants was consistent with that of Fv/Fm (Figure 8H). These results further supported that overexpression of *MdCCX1* in roots enhanced the salt tolerance of apple plants.

### *MdCCX1*-Overexpression Enhanced Apple Plant Salt Tolerance by Inhibiting Excessive Accumulation of Na^+^ and Reactive Oxygen Species

**FIGURE 9:**
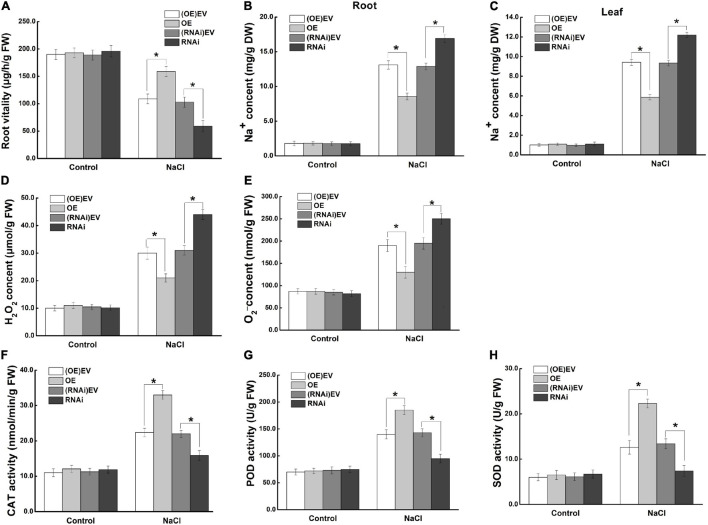
*MdCCX1* regulated Na^+^ accumulation and antioxidant enzymes activities. **(A)** Root vitality. **(B)** Na^+^ content in roots. **(C)** Na^+^ content in leaves. Hydrogen peroxide (H_2_O_2_) content **(D)** and superoxide anion (O_2_^–^) content **(E)** in transgenic apple roots. **(F–H)** Enzymatic activity of antioxidant enzymes CAT **(F)**, POD **(G)**, and SOD **(H)** under normal and NaCl stress conditions. The values of each index are the average values of all lines in transgenic plants of the same type. Values are means of 20 replicates ± SD. * in each panel indicates values that are significantly different from the corresponding control lines at *p* < 0.05, using Student’s *t-test*.

To investigate the mechanism of *MdCCX1* in enhancing apple plant salt tolerance, we further focus on the effect of *MdCCX1*-overexpression in roots, as the first plant organ affected by soil-salinity. Measurement of root activity showed no difference between *MdCCX1* transgenic and control lines under normal conditions. After salt treatment, the root vitality of MdCCX1-OE lines was significantly higher, whereas that of the MdCCX1-RNAi lines was significantly lower than the control lines (Figure 9A). This indicated that overexpression of *MdCCX1* alleviated the damage caused by salt stress to apple roots. Based on the function of MdCCX1 in inhibiting Na^+^ accumulation in yeast and plants, we measured the Na^+^ content in these apple plants. As we expected, the Na^+^ content in roots of MdCCX1-OE lines was significantly lower than that of control after salt treatment, and the MdCCX1-RNAi lines exhibited the opposite results (Figure 9B). Moreover, the Na^+^ content in leaves of these plants also exhibited the same result, suggesting that *MdCCX1*-overexpression in apple roots could also reduce the Na^+^ accumulation in leaves under salt stress (Figure 9C). These results suggested that *MdCCX1*-overexpression could enhance the salt tolerance of apple plants by inhibiting excessive Na^+^ accumulation.

To further investigate the function of MdCCX1 in promoting ROS scavenging in apple plants under salt stress, we measured the ROS accumulation and antioxidant enzymes’ activity in the roots of these plants. Under normal conditions, there was no difference between different lines of apple plants. Under salt stress, the H_2_O_2_ and O_2_^–^ contents were significantly lower (Figures 9D,E), but the activity of antioxidant enzymes (CAT, POD, SOD) was significantly higher in roots of MdCCX1-OE lines. The opposite trend was observed in the MdCCX1-RNAi lines (Figures 9F–H). These results indicated that *MdCCX1*-overexpression promoted ROS elimination under salt stress, thus reducing stress damage caused by excessive ROS accumulation. In all, MdCCX1 plays a positive role in apple plant salt tolerance by inhibiting excessive accumulation of Na^+^ and ROS under salt stress.

## Discussion

Abiotic stress resulting from excessive salinity has damaging consequences for plant growth, development, and crop productivity. With the development of biotechnology, it has become a mature and fast way to use the transgenic method to develop resistant crops, which depends on the functional characterization of stress-related genes. To date, some work had revealed that CCXs play a vital role in abiotic stress responses. However, no detailed functional studies on CCX family proteins in apple have been reported. Here, we cloned the *MdCCX1* gene from apple and identified its function in regulating salt tolerance using transgenic *Arabidopsis* and apple plants. These results, combined with the subcelluar localization of MdCCX1 and its function in inhibiting Na^+^ accumulation, let us to conclude that MdCCX1 acts as a plasma membrane-located Na^+^ efflux transporter to enhance plant salt tolerance.

### Subcellular Localization Play Vital Roles in the Function of Ion Transporters

The subcellular localization was one of the basic characteristics of ion transporters which may directly affect their function. The *Arabidopsis* cell membrane-located Na^+^/H^+^ antipoters, such as SOS1, reduce the [Na^+^]_cyto_ by extruding Na^+^ from the cytoplasm (Shi et al., 2000; Song et al., 2012). On the other hand, the Na^+^/H^+^ antiporters on the tonoplast (e.g., AtNHX1) segregate Na^+^ into the vacuole to reduce the [Na^+^]_cyto_ (Sottosanto et al., 2007; Asif et al., 2011; Kumar et al., 2017). Overexpression of either of these two kinds of Na^+^/H^+^ antiporters can enhance salt tolerance in transgenic plants. As to CCX proteins, AtCCX2 is located on the ER membrane and regulates plant salt tolerance by maintaining Ca^2+^ and Na^+^ homeostasis (Corso et al., 2018). AtCCX1 is located on the tonoplast in yeast cells and increases Na^+^ accumulation in yeast vacuole, suggesting it may be a tonoplast-located Na^+^ transporter (Chen et al., 2011), but experimental proof in plants is lacking. Studies on CaCA family proteins have found proteins that showed different localization in yeast and plant cells, such as AtNCL (Wang et al., 2012). In rice, OsCCX2 was proved to be located on the plasma membrane and mediates the efflux of Cd^2+^ to enhance Cd^2+^ tolerance (Hao et al., 2018). These studies indicate different subcellular localization patterns and ion transport activities of different CCX family proteins in different plant species. Because of this, we firstly investigated the subcellular localization of MdCCX1 before identifying its ion transport characteristics. Based on the fluorescence observation results of MdCCX1-GFP fusion protein in transgenic tobacco and *Arabidopsis* protoplast cells, we proved that the MdCCX1 is a plasma membrane-located protein (Figure 2). This results differs from AtCCX1 and AtCCX2, but agrees with OsCCX2 and AtSOS1.

### The Ion Transport Properties of MdCCX1 in Yeasts

Sequence comparison and phylogenetic analysis showed that MdCCX1 was an ortholog of AtCCX1 in apple (Figure 1). Studies on the identification of ion transport activity of CCX family proteins using K667 yeast mutant have shown that, as CaCA family members, CCX proteins can transport Ca^2+^ and other ions. Thus, we firstly identified the ion transport capacity of MdCCX1 using the K667 yeast mutant. Unexpectedly, MdCCX1 did not suppress the sensitivity of K667 to high Ca^2+^, nor did it suppress the sensitivity to other ions (Ba^2+^, Cu^2+^, Mg^2+^, Li^+^, Mn^2+^, and Cd^2+^) (Figure 3 and Supplementary Figures 1A,B).

The expression of *MdCCX1* was strongly induced by NaCl treatment (Figure 1D), suggesting that it may participate in the salt response of apple plants. Therefore we further identified the Na^+^ transport capacity of MdCCX1 using two Na^+^-sensitive yeast mutants, which are the single mutant yeast strain Δ*ena1-4* that lacks ENA and the double mutants yeast strain Δ*ena1-4*, Δ*nha1* that lacks ENA and NHA on the cell membrane. Their phenotype of sensitivity to high Na^+^ made these two strains more suitable than K667 for this study. Encouragingly, *MdCCX1*-overexpression significantly inhibited the sensitivity of both yeast mutant strains to high Na^+^ (Figures 3B,C), which was further supported by the growth curves of these strains under NaCl treatment (Figures 3D,E). Because the ENA in yeast was a P-type ATPases responsible for excreting Na^+^ and selectively absorbing K^+^, for further studying the mechanism of MdCCX1 in enhancing yeast salt tolerance, we overexpressed MdCCX1 in the R5421 yeast mutant to rule out the effect of K^+^ (Supplementary Figure 1C). These results, together with the lower Na^+^ content in *MdCCX1*-transformed yeast cells under NaCl treatment (Figure 3F), suggest that MdCCX1 enhances the salt tolerance by inhibiting Na^+^ accumulation in yeast cells, just like the SOS1 protein in plants.

### MdCCX1 Enhances Plant Salt Tolerance by Inhibiting the Excessive Accumulation of Na^+^

As members of the CaCA superfamily, CCX proteins play important roles in regulating plant growth and stress response by affecting homeostasis and accumulation of various ions. However, studies on the function and stress signaling of CCX proteins in plants are scarce, and only the AtCCX2 in *Arabidopsis* has been shown to be involved in the regulation of salt tolerance by affecting Ca^2+^ homeostasis (Corso et al., 2018). Ion transporters mediate plant salt tolerance mainly by transporting Na^+^ out of the cell (plasma membrane-localized transporters) or isolating Na^+^ in the vacuole (tonoplast-localized transporters), thereby reducing the Na^+^ content in the cytosol. However, no studies have shown that CCX proteins can directly regulate Na^+^ accumulation to regulate salt tolerance in plants. In this study, we found that MdCCX1 located on the plasma membrane of plant cells (Figure 2) and inhibited the Na^+^ accumulation in yeast (Figure 3). These results suggested that MdCCX1 may have the function to inhibit Na^+^ accumulation in plants, thus leading to enhanced salt tolerance, just like SOS1. In *Arabidopsis*, AtSOS1 encoded a plasma membrane Na^+^/H^+^ antiporter that plays a critical role in Na^+^ extrusion from the cytosol into the apoplast or back to the soil solution and in controlling long distance Na^+^ transport from the root to shoot (Shi et al., 2002; Oh et al., 2010). We transformed *MdCCX1* into *Arabidopsis*, apple calli, and apple plant roots to confirm this hypothesis and identified the influence of *MdCCX1*-overexpression on plant salt tolerance. Based on the phenotypic comparison and stress-related physiological indicators measurement, we proved that overexpression of *MdCCX1* significantly improved the salt tolerance of the transgenic materials (Figures 4–8). Besides, the apparent lower Na^+^ content in these transgenic materials suggested that *MdCCX1*-overexpression significantly inhibited the excessive accumulation of Na^+^ under salt stress (Figures 6, 7, 9). These results indicate that, as we excepted, MdCCX1 enhances plant salt tolerance by inhabiting Na^+^ accumulation.

### MdCCX1 Enhances Plant Salt Tolerance by Promoting Reactive Oxygen Species Scavenging

Salt stress-caused ROS production is used as a physiological index of stress damage. Besides, excessive accumulation of ROS in plant tissues leads to oxidative damage, detrimental to plant growth and development. In this study, the ROS was induced significantly by salt treatment in *Arabidopsis* and apple plants (Figures 5, 9 and Supplementary Figure 3). The significantly lower ROS accumulation in *MdCCX1*-overexpression transgenic plants (Figures 5, 9 and Supplementary Figure 3) suggested that these plants suffered less stress damage caused by high Na^+^. This result can be explained largely by the significant reduction in Na^+^ content in these transgenic plants (Figures 6, 8). However, the significantly higher activity of antioxidant enzymes in these *MdCCX1*-overexpression transgenic materials indicates that there might be another reason for the lower ROS under salt stress. Compared with control, the activity of the antioxidant enzymes CAT, POD, and SOD was significantly higher in the *MdCCX1*-overexpression transgenic *Arabidopsis*, apple calli and apple plants (Figures 6, 7, 9), suggesting that MdCCX1 could promote the activity of these antioxidant enzymes under salt stress, and thus leaded to lower ROS accumulaion and less oxidative damage. These results suggested that, in addition to inhibiting Na^+^ accumulation, MdCCX1 could also enhance plant salt tolerance by promoting ROS scavenging. This speculation is consistent with several other studies of plant CCX proteins (Li et al., 2016; Yan et al., 2020).

**FIGURE 10:**
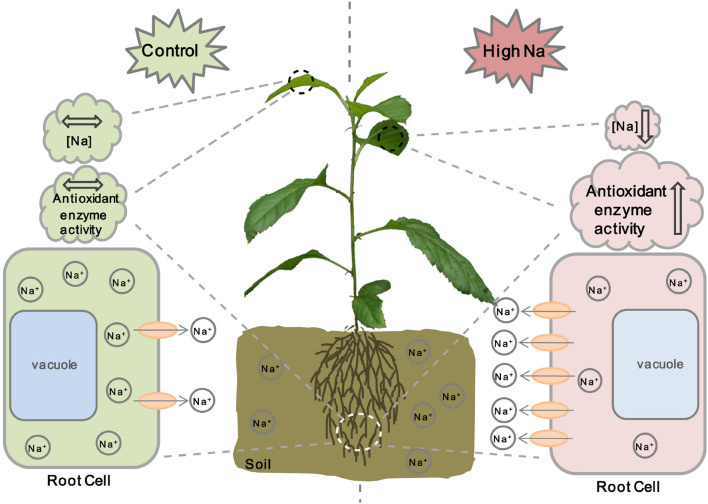
A working model illustrating MdCCX1-mediated salt response in apple plants. Under normal conditions, the expression level of *MdCCX1* is maintained at a basal level to maintain ion homeostasis and normal plant growth and development. Under salt stress, the *MdCCX1* expression is induced, and the MdCCX1 protein accumulates in roots of apple plants. The accumulated MdCCX1 protein inhibits the accumulation of Na^+^ in roots by expelling excess Na^+^ into the extracellular space or rhizosphere environment. This function of MdCCX1 may also facilitate the transport and dispersion of Na^+^ to other tissues. Moreover, with the continues of salt stress, the increase of Na^+^ content induces MdCCX1 accumulation in both roots and leaves, and the accumulated MdCCX1 in turn promotes the activity of antioxidant enzymes to scavenge ROS. The ovals represent the MdCCX1 transporters, and the arrows represents the direction of Na^+^ transport across the cell membrane.

Based on the results obtained in this study and previous studies on other ion transporters related to salt stress, we propose a model for MdCCX1 regulation of salt tolerance in apple plants (Figure 10). Under normal conditions, the expression level of *MdCCX1* is maintained at a basal level to maintain ion homeostasis and normal plant growth and development. When plants are exposed to salt stress conditions, *MdCCX1* expression in roots will be induced by NaCl quickly and significantly. Then the accumulated MdCCX1 protein could inhibit the excessive accumulation of Na^+^ in roots by mediating its efflux from the cytosol into the apoplast. This function of MdCCX1 may also facilitate the transport and dispersion of excessive Na^+^ in roots to other plant tissues and its removal from the plant by other means. On the other hand, the increase of Na^+^ content in plants induces MdCCX1 accumulation in both roots and leaves. The accumulated MdCCX1 promotes the ROS scavenging by promoting the activity of antioxidant enzymes, thereby reducing oxidative damage caused by excessive ROS accumulation under salt stress. Further studies are needed to explore how MdCCX1 promotes the antioxidant enzymes’ activities under salt stress.

## Data Availability Statement

All data supporting the findings of this study are available within the paper and within its supplementary data published online.

## Author Contributions

JY, KM, and FM conceived the project and wrote the manuscript. JY and KM designed the research plan. JY, WL, XG, PC, and YC carried out the experiments. JY, WL, XG, and KM analyzed the data. All authors read and approved of the content.

## Conflict of Interest

The authors declare that the research was conducted in the absence of any commercial or financial relationships that could be construed as a potential conflict of interest.

## Publisher’s Note

All claims expressed in this article are solely those of the authors and do not necessarily represent those of their affiliated organizations, or those of the publisher, the editors and the reviewers. Any product that may be evaluated in this article, or claim that may be made by its manufacturer, is not guaranteed or endorsed by the publisher.

## Footnotes

1
https://www.arabidopsis.org/

2
http://smart.embl-heidelberg.de

3
http://ncbi.nlm.nih.gov/Structure/cdd/wrpsb.cgi

4
https://www.rosaceae.org/

## Supplementary Material

Supplementary Figure 1 Tolerance of yeast transformants expressing *MdCCX1* or the pDR196 to different metal ions. **(A)** Comparison of tolerance of K667 yeast strains expressing *MdCCX1* or the pDR196 to Li^+^, Mn^2+^, Cd^2+^, Ba^2+^, Cu^2+^, or Mg^2+^. **(B)** Phenotypes of R5421 yeast strains expressing *MdCCX1* or the pDR196 grown in AP medium supplemented with different concentrations of K^+^. Pictures were captured after 3 days of growth at 28°C. Two positive transformets of each type were used in the experiment.

Supplementary Figure 2 Identification of transgenic *Arabidopsis* and calli. **(A)** PCR identification of the *MdCCX1* transgene based on genomic DNA extracted from *Arabidopsis* plants. Genomic DNA extracted from “Col” plants and H_2_O were used as negative controls, and the *MdCCX1*-pBI121 vector plasmid was used as a positive control. **(B)** Expression level of *MdCCX1* in transgenic *Arabidopsis* plants. *AtActin* served as an internal reference gene. **(C)** PCR identification of the *MdCCX1* transgene based on genomic DNA extracted from apple calli. Genomic DNA extracted from WT calli and H_2_O were used as negative controls, and the *MdCCX1*-pBI121 vector plasmid was used as a positive control. **(D)** Expression level of *MdCCX1* in transgenic apple calli. *MdMDH* served as an internal reference gene. Bars labeled with different letters in each panel are significantly different (*p* < 0.05, one-way ANOVA analysis and Duncan’s test).

Supplementary Figure 3 Histochemical staining of apple leaves. **(A)** Histochemical staining of hydrogen peroxide (H_2_O_2_) in apple leaves using NBT (nitro blue tetrazolium). **(B)** Histochemical staining of superoxide anion (O_2_^–^) in apple leaves using DAB (3,3′-diaminobenzidine). Scale bars, 3 cm.
