# Supplementary figures and images for: Cation/Ca2+ Exchanger 1 (MdCCX1), a Plasma Membrane-Localized Na+ Transporter, Enhances Plant Salt Tolerance by Inhibiting Excessive Accumulation of Na+ and Reactive Oxygen Species
Source: Front Plant Sci. 2021 Oct 13;12:746189. doi: 10.3389/fpls.2021.746189 (PMC8549818; doi:10.3389/fpls.2021.746189)

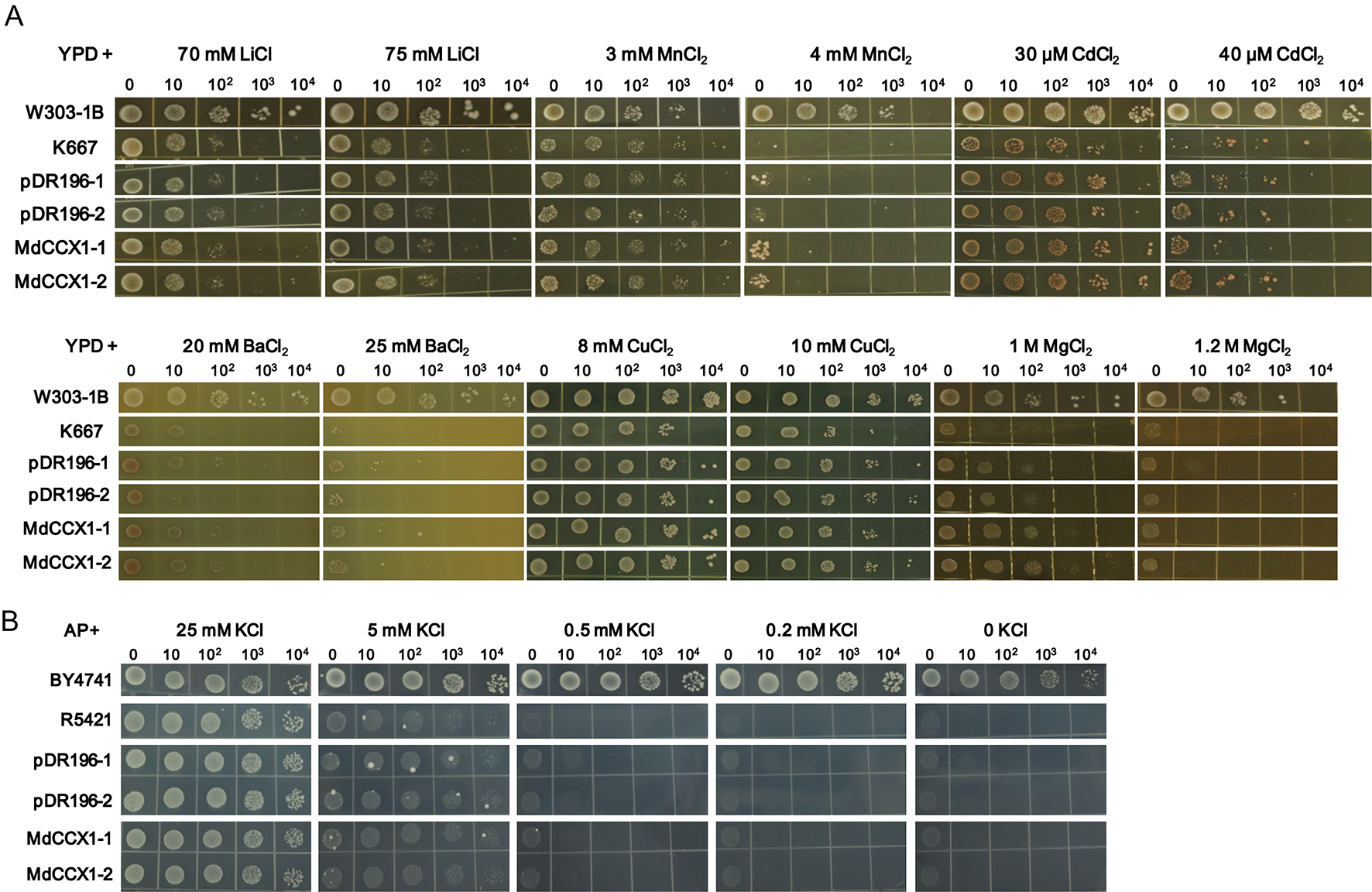

Supplement: Supplementary Figure 1 — Tolerance of yeast transformants expressing MdCCX1 or the pDR196 to different metal ions. (A) Comparison of tolerance of K667 yeast strains expressing MdCCX1 or the pDR196 to Li+, Mn2+, Cd2+, Ba2+, Cu2+, or Mg2+. (B) Phenotypes of R5421 yeast strains expressing MdCCX1 or the pDR196 grown in AP medium supplemented with different concentrations of K+. Pictures were captured after 3 days of growth at 28°C. Two positive transformets of each type were used in the experiment. [file Image_1.JPEG]

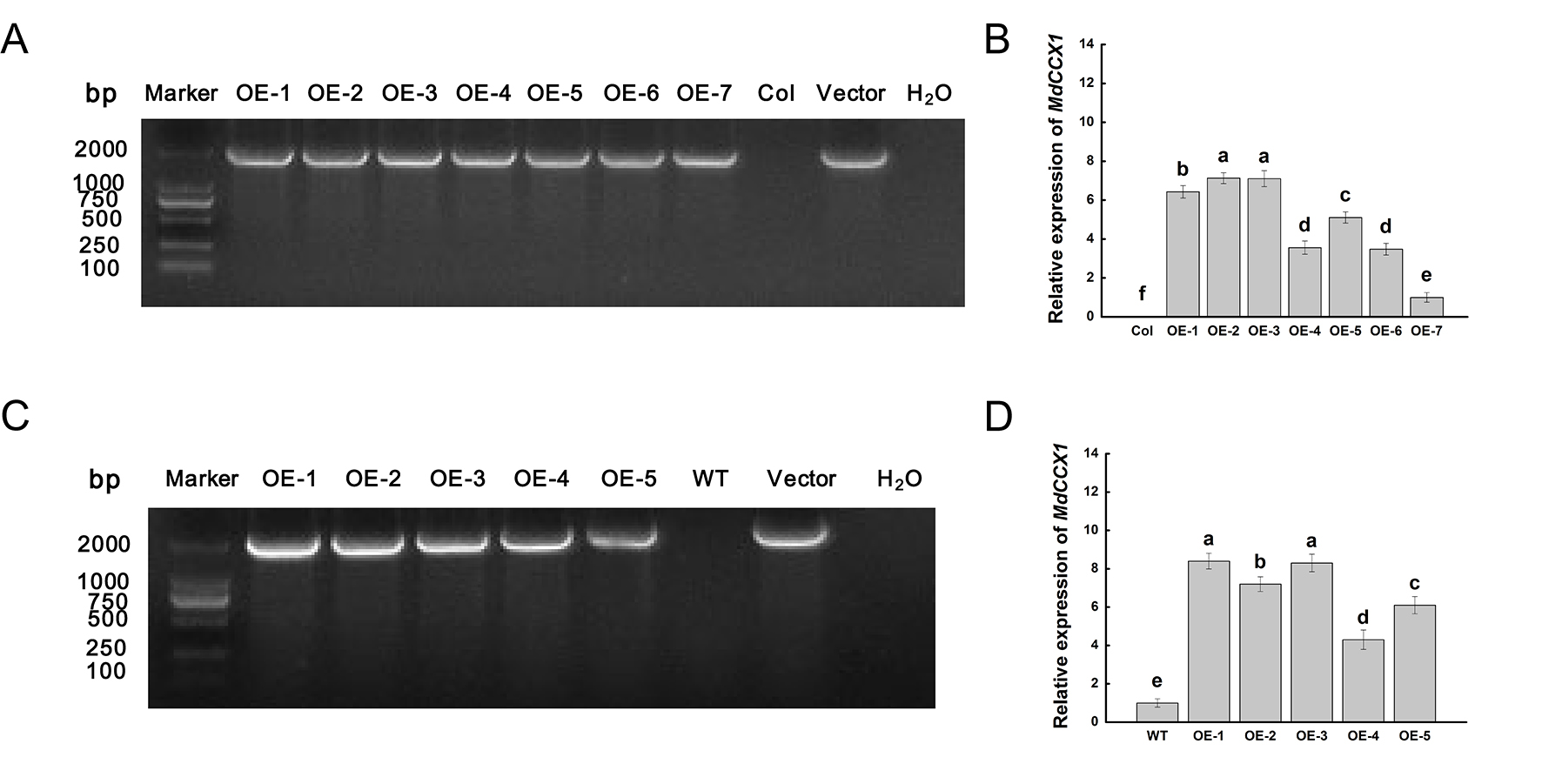

Supplement: Supplementary Figure 2 — Identification of transgenic Arabidopsis and calli. (A) PCR identification of the MdCCX1 transgene based on genomic DNA extracted from Arabidopsis plants. Genomic DNA extracted from “Col” plants and H2O were used as negative controls, and the MdCCX1-pBI121 vector plasmid was used as a positive control. (B) Expression level of MdCCX1 in transgenic Arabidopsis plants. AtActin served as an internal reference gene. (C) PCR identification of the MdCCX1 transgene based on genomic DNA extracted from apple calli. Genomic DNA extracted from WT calli and H2O were used as negative controls, and the MdCCX1-pBI121 vector plasmid was used as a positive control. (D) Expression level of MdCCX1 in transgenic apple calli. MdMDH served as an internal reference gene. Bars labeled with different letters in each panel are significantly different (p < 0.05, one-way ANOVA analysis and Duncan’s test). [file Image_2.JPEG]

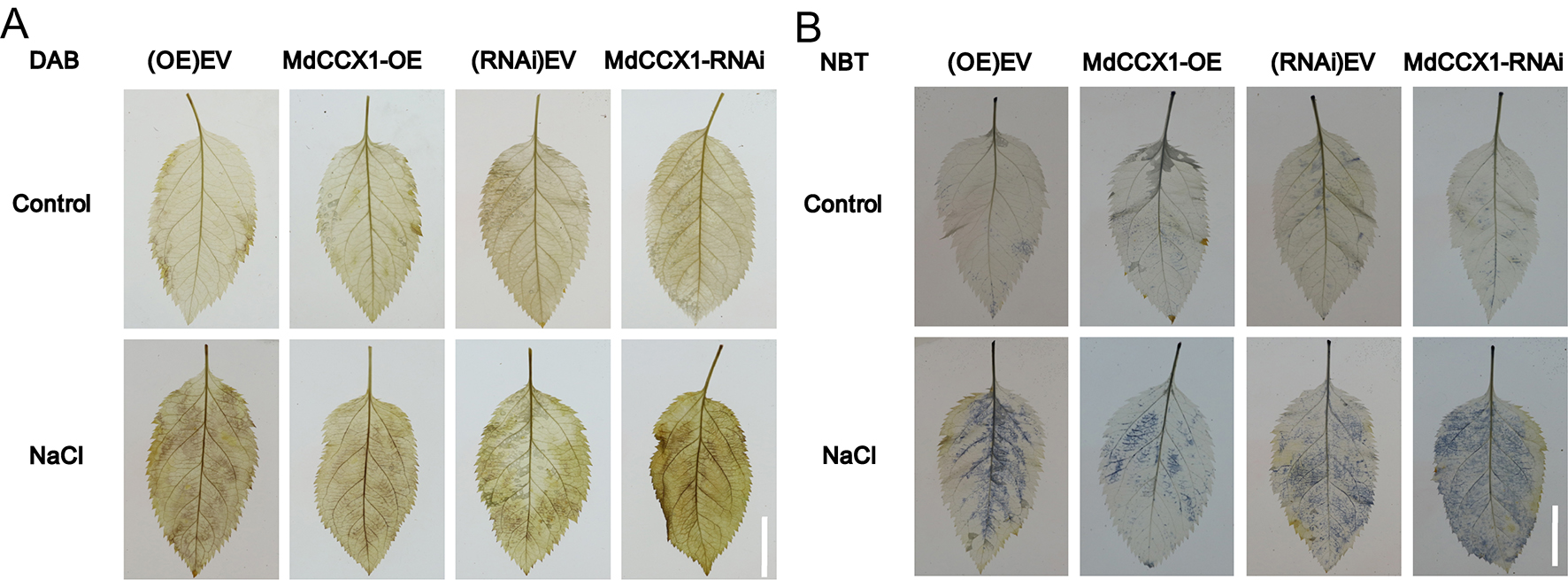

Supplement: Supplementary Figure 3 — Histochemical staining of apple leaves. (A) Histochemical staining of hydrogen peroxide (H2O2) in apple leaves using NBT (nitro blue tetrazolium). (B) Histochemical staining of superoxide anion (O2–) in apple leaves using DAB (3,3′-diaminobenzidine). Scale bars, 3 cm. [file Image_3.JPEG]
