# Supplementary material for: Cation/Ca2+ Exchanger 1 (MdCCX1), a Plasma Membrane-Localized Na+ Transporter, Enhances Plant Salt Tolerance by Inhibiting Excessive Accumulation of Na+ and Reactive Oxygen Species
Source: Front Plant Sci. 2021 Oct 13;12:746189. doi: 10.3389/fpls.2021.746189 (PMC8549818; doi:10.3389/fpls.2021.746189)
Supplement: Supplementary file 4 [file Table_1.DOCX]

Table S1. Primers used in this study.

| **Annotation** | **Primer name** | **Sequence (5'-3')** |
| --- | --- | --- |
|  |  |  |
| **Vector construction** | MdCCX1-PMD-F | ATGGCAAGCTCAACCTCC |
|  | MdCCX1-PMD-R | TCAAAAGAACAAGAGCCT |
|  | MdCCX1-2300GFP-F | CGAGCTCGGTACCCGGGGATCCATGGCAAGCTCAACCT |
|  | MdCCX1-2300GFP-R | CCTTGCTCACCATGGTGTCGACAAAGAACAAGAGCCTG |
|  | MdCCX1-pBI121-F | ACGGGGGACTCTAGAGGATCCATGGCAAGCTCAACCT |
|  | MdCCX1-pBI121-R | CGATCGGGGAAATTCGAGCTCTCAAAAGAACAAGAGC |
|  | MdCCX1-pDR196-F | TCCCCCGGGCTGCAGGAATTCATGGCAAGCTCAACCT |
|  | MdCCX1-pDR196-R | GGGCCCCCCCTCGAGGTCGACTCAAAAGAACAAGAGC |
|  | MdCCX1-RNAi-F | GGGGACAAGTTTGTACAAAAAAGCAGGCTTCATGGCAAGCTCAACCTCC |
|  | MdCCX1-RNAi-R | GGGGACCACTTTGTACAAGAAAGCTGGGTTAAATATTTTCAGATAATG |
|  |  |  |
| **qRT-PCR** | MdCCX1-qF | GAGGAAGGAGCTGCACAAGGC |
|  | MdCCX1-qR | GTCACTGAAGCAACTGCATATGGC |
|  | MdMDH-qF | CGTGATTGGGTACTTGGAAC |
|  | MdMDH-qR | TGGCAAGTGACTGGGAATGA |
|  | AtActin-qF | GGAAAGGATCTGTACGGTAAC |
|  | AtActin-qR | TGTGAACGATTCCTGGAC |
|  | AtSOD1-qF | CTAATTGGGATTGACGTGTGGGAGC |
|  | AtSOD1-qR | GAAGACGTCCGCTGCATATTTCCAG |
|  | AtPOD-qF | TAGGGTCGGGTAAACCTGTG |
|  | AtPOD-qR | GTTGCTCTCTCCGGTGGTC |
|  | AtCAT1-qF | AACTTCCCTGTATTCTTCGTCCGTG |
|  | AtCAT1-qR | TGGGATTCGGTTTCAATGCATGGAC |
|  |  |  |
